# Development of Ultrasonic Guided Wave Transducer for Monitoring of High Temperature Pipelines

**DOI:** 10.3390/s19245443

**Published:** 2019-12-10

**Authors:** Anurag Dhutti, Saiful Asmin Tumin, Wamadeva Balachandran, Jamil Kanfoud, Tat-Hean Gan

**Affiliations:** 1 Brunel University London, Kingston Ln, Uxbridge, Middlesex UB8 3PH, UK; 2 TWI Lt, Granta Park, Cambridge CB21 6AL, UK

**Keywords:** gallium phosphate, piezoelectric wafer active sensor, thickness shear, high-temperature monitoring, ultrasonic, guided wave, structural health monitoring

## Abstract

High-temperature (HT) ultrasonic transducers are of increasing interest for structural health monitoring (SHM) of structures operating in harsh environments. This article focuses on the development of an HT piezoelectric wafer active sensor (HT-PWAS) for SHM of HT pipelines using ultrasonic guided waves. The PWAS was fabricated using Y-cut gallium phosphate (GaPO_4_) to produce a torsional guided wave mode on pipes operating at temperatures up to 600 °C. A number of confidence-building tests on the PWAS were carried out. HT electromechanical impedance (EMI) spectroscopy was performed to characterise piezoelectric properties at elevated temperatures and over long periods of time (>1000 h). Laser Doppler vibrometry (LDV) was used to verify the modes of vibration. A finite element model of GaPO_4_ PWAS was developed to model the electromechanical behaviour of the PWAS and the effect of increasing temperatures, and it was validated using EMI and LDV experimental data. This study demonstrates the application of GaPO_4_ for guided-wave SHM of pipelines and presents a model that can be used to evaluate different transducer designs for HT applications.

## 1. Introduction

There is an increasing emphasis on the development of structural health monitoring (SHM) systems using in situ sensors that can inform an operator about the health of their critical infrastructure, develop damage and estimate the remaining useful life. Piezoelectric wafer active sensors (PWASs) are increasingly used for many SHM applications, as they are lightweight, inexpensive and can be permanently installed on structures. They have been successfully demonstrated on various critical components and substructures in many industrial applications [1]. PWASs can be used for damage detection in three different ways: guided-wave ultrasonics [2], high-frequency modal sensing [3] and acoustic emission passive detection [4]. SHM applications for assets subjected to an extreme operational environment such as high temperature (HT) are in great demand to monitor critical components in gas turbines and nuclear power industry. This has led to rapid developments in the field of HT piezoelectric sensing. Several accelerometers, surface acoustic wave sensors, ultrasonic transducers and pressure sensors for temperatures up to 1250 °C have been reported [5].

High-temperature pipelines (HTPs) are critical components in nuclear power plants, providing connections between feed water pumps, a boiler or heat exchanger and a turbine. HTPs are operating at extreme temperatures up to around 600 °C [6], high pressures and subjected to continuous cyclic loadings. This can lead to the development of creep-fatigue- [7], corrosion- [8] and stress corrosion cracking-type [9] defects. Currently, they undergo periodic inspections during maintenance outages, but new technologies are being sought after for in situ monitoring and early warnings of material degradation [10]. A number of SHM technologies have been developed for pipelines [11]. A suitable solution is demonstrated by the ultrasonic guided wave (UGW) technique, as it provides full cross-sectional area coverage for a range of tens of meters from a single location. Rings of shear transducers are used to transmit unidirectional and axially symmetric ultrasonic wave modes [12] in a frequency range of 20–100 kHz, and they can detect the presence of structural defects such as cracks and corrosion by analysing reflected waves. Commercial products such as gPIMS [13] are now available for SHM of pipelines but are limited for continuous operation at temperatures below ~100 °C. To enable guided-wave SHM for HTPs, piezoelectric transducers that can perform reliably at HT are required.

## 2. High-Temperature Ultrasonic-Guided-Wave Transducers

**Figure 1:**
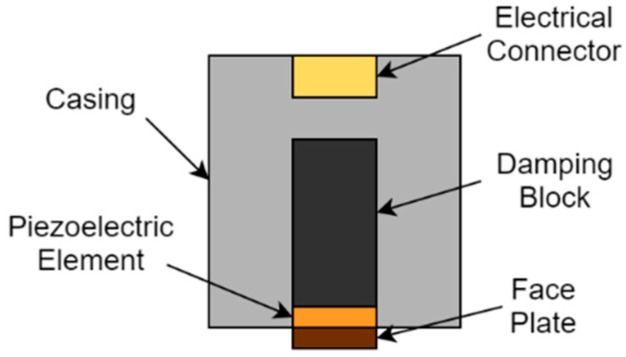
Construction of a generic piezoelectric transducer.

Several transduction technologies based on piezoelectric, electromagnetic-acoustic and magnetostrictive principles have been developed for the generation and the detection of UGWs. For application at HT, piezoelectric sensing is the most promising technique due to its thermal stability and reliability [5]. A thickness-shear (TS) piezoelectric transducer consists of five main components: a face plate, a piezoelectric element, a backing/damping block, a casing and electrical connections, as illustrated in Figure 1. The electrical connection allows for the transfer of an electrical pulse to and from the piezoelectric element. The casing provides mechanical support for the whole transducer as well as electrical shielding for the piezoelectric element. The damping or backing block damps the vibration of the piezoelectric element, after the excitation pulse is transmitted. The piezoelectric element generates a mechanical signal from an electrical input or, conversely, an electrical signal from a mechanical input, enabling transmission and reception of ultrasonic signals, respectively. The face plate protects the fragile piezoelectric element and is used for mechanical impedance matching.

In pipes, there is a large number of wave modes present in the frequency range of interest, and a transducer can excite all the modes that exist within its frequency bandwidth. This can complicate the data and make its interpretation challenging. To simplify the guided wave response, transducers are designed to minimise the excitation of unwanted modes and achieve a linear frequency response in the region of operation to avoid mode coupling, frequency jumps and performance dips. For the HT SHM application, transducers must also provide sufficient ultrasonic outputs at the target temperature and sustain their electromechanical response over prolonged periods of time to achieve the desired defect sensitivity of the SHM system. This requires careful selection of piezoelectric material, understanding of its thermal characteristics and compatibility with other transducer subcomponents.

### 2.1. High-Temperature Piezoelectric Materials

Piezoelectric materials are characterised by a number of interrelated coefficients standardised by the IEEE [14,15], which define their piezoelectric, dielectric and elastic behaviour. These coefficients are used to describe the coupling between structural and electrical behaviour of the material. It can be expressed as a relation between the material stress and its permittivity at a constant stress (stress charge form, Equation (1)) or as a relation between the material strain and its permittivity at a constant strain (strain charge form, Equation (2)).

(1) $$\mathrm{Stress}\;\mathrm{charge}\;\mathrm{form}:\;T=c^{E}S-e^{T}E,\;D=eS+\epsilon^{S}E$$

(2) $$\mathrm{Strain}\;\mathrm{charge}\;\mathrm{form}:\;S=s^{E}T+d^{T}E,\;D=dT+\epsilon^{T}E$$

where E is the electric field, D is the electric displacement, S is the mechanical strain, and T is the stress. The material parameters $s^{E}$, d and $\epsilon^{T}$ are material compliance, coupling property and relative permittivity at a constant stress, respectively, and they are tensors of ranks 4, 3 and 2, respectively, and represented using the abbreviated subscript Voigt notation.

A useful guide for the selection of an appropriate piezoelectric material is its figure of merit (FOM), which is defined based on a specific application [16]. A key FOM for an HT transducer is the product of piezoelectric charge coefficient d and voltage coefficient g, where a higher value gives a higher electromechanical coupling coefficient, k, which is defined as the ratio of stored electrical energy to applied mechanical energy and vice versa.

Several new high-performance HT piezoelectric materials have been developed [17,18,19], and they can be broadly categorized into two types: ferroelectric polycrystalline (such as lead zirconate titanate (PbZrTiO_3_ and barium titanate (BaTiO_3_)) and single crystals (such as lithium niobate (LiNbO_3_) and quartz (SiO_2_)). Ferroelectric materials possess high FOMs (e.g., *k* = 0.7 for PbZrTiO_3_) and are the most commonly used piezoelectric materials for ultrasonic transducers. However, they are limited to operate at temperatures below their Curie temperatures ($T_{c}$). Beyond $T_{c}$, they depolarize, i.e., their ferroelectric phase transforms into a high-symmetry nonferroelectric phase, and are no longer piezoelectric. Ferroelectric materials at temperatures exceeding 0.5$T_{c}$ [20] can undergo accelerated thermal ageing and property degradation. They also exhibit pyroelectric behaviour, which can lead to instability of their electromechanical properties at HT. Nonferroelectric single crystal materials are being increasingly explored, as they do not undergo phase transition or domain-related aging behaviour, have high resistivity and low losses and can provide excellent thermal stability. Various types of single crystals have been investigated for HT applications, and monoclinic-type BiB_3_O_6_ (BIBO) and langasite-type Ca_3_TaGa_3_Si_2_O_14_ (CTGS) trigonal crystals have shown stable piezoelectric properties at temperatures up to 700 and 900 °C, respectively [21].

Gallium orthophosphate (GaPO_4_) was selected for this study, as it offers high electrical resistivity, low acoustic losses, no pyroelectricity and thermally stable properties (constant value of $d_{11}$ at temperatures up to 700 °C [22]), which makes it a suitable candidate for HT UGW transducers. GaPO_4_ crystals belong to the trigonal 32-point group and can be synthesised using a hydrothermal process. The AVL (now Piezocryst Advanced Sensorics GmbH) developed the first commercial product GM12D, which was an uncooled pressure sensor for combustion engines, and since then, GaPO_4_ has been explored for a large field of HT resonator applications including temperature-compensated cuts for bulk acoustic wave (BAW) [23], surface acoustic wave (SAW) [24] and microbalance [25] operating at temperatures up to 720 °C. GaPO_4_ resonators can be obtained in different crystal cuts and depending on crystal cut angles, and their sensitivity, resolution and linearity differ in a wide range. Singly rotated Y-cut GaPO_4_ resonators with different angles have been investigated [26]. Y −84° was shown to have a very high Q factor and a high drive level dependency appropriate for pressure sensing applications, whilst Y +27° displayed a linear resonance frequency dependence on temperature [27], which makes it suitable for temperature sensing. An X-cut resonator was investigated for phased arrays and displayed encouraging results for SHM applications [28].

### 2.2. High-Temperature Transducer Design

The selection of transducer passive materials including electrodes, matching layers, backing materials, connecting wires and adhesives also requires careful consideration. The coefficient of thermal expansion (CTE) of the piezoelectric material should be compatible with the structure and the materials within the particular transducer assembly. Excessive differential thermal expansion can lead to permanent damage to the transducer assembly or thermal stresses within the piezoelectric material, leading to the generation of unwanted modes of vibration. Bonding of different materials within the transducer assembly is also challenging, as the temperature required for bonding must be below $T_{c}/2$ and pressure used in bonding could crack the piezoelectric material. The bond must also remain strong at the operating temperature of the device and be elastic in order to be able to change shape, as the piezoelectric deforms and the other parts of the transducer expand or contract due to changes in temperature.

Common methods of bonding include the use of HT epoxy, which is a thermosetting polymer used as a glue. Additionally, glass solder can be used at temperatures up to 500 °C, as for higher temperatures, it reacts chemically with other components. Other methods include regular soldering, diffusion bonding, ultrasonic welding, cementing, sol-gel and vacuum brazing. A comprehensive review of these bonding techniques and designs of ultrasonic transducers at HT can be found in the literature [17]. Unlike bulk transducers that require lower internal damping to achieve resonant transducers, piezoelectric materials must be damped heavily to produce broadband, guided-wave transducers.

### 2.3. Thickness-Shear Piezoelectric Material Characterisation

**Figure 2:**
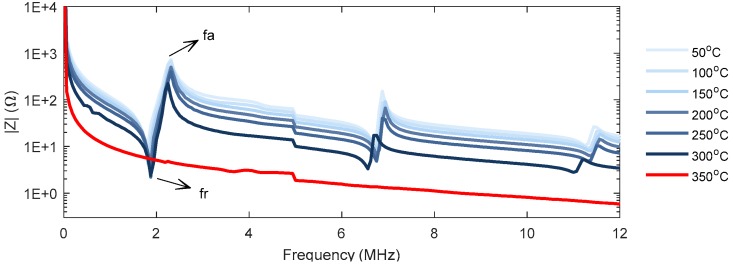
Example of high-temperature electromechanical impedance (EMI) response from a Lead zirconate titanate (PZT) plate.

It is critical to characterise piezoelectric properties for the transducer design and modelling and also to analyse their performance for the application under study. Not all the parameters are usually available from datasheets, the real values may deviate from specified values due to electrodes and polarisation, or they might not be available for the range of the temperature of interest. The material properties can be derived from their electromechanical impedance (EMI) measurements in accordance with BS EN 50324-2:2002 [29]. The expression of the EMI of a plate vibrating in a TS mode as a function of frequency can be shown as:

(3) $$z\left(\omega \right)=\frac{t}{i\omega \epsilon_{22}^{S}A}\left[1-k^{2}\frac{\tan\left(\frac{\omega }{4f_{a}}\right)}{\left(\frac{\omega }{4f_{a}}\right)}\right],$$

where t is the plate of thickness t and A is the electrode area. The EMI response represents the dynamic behaviour of the piezoelectric material, when an AC voltage is applied. An example of the EMI response of a commonly used PZT material is presented in Figure 2.

The electromechanical resonances are associated with the reaction of a ceramic body to an ultrasound. EMI versus frequency plots can be presented on a logarithmic scale to emphasise weaker excitable modes including a harmonics, spurious or unwanted mode. For piezoelectric materials in class 32, the TS mode of a vibration can be obtained using Y-cut plates with electrode surfaces on a plane normal to the $x_{2}$ direction. For the TS mode, the eigenfrequency equation for the harmonics is given as:

(4) $$f_{n}=n\frac{v}{2h},\;v=\sqrt{\frac{c_{66}^{D}}{\rho }},$$

where $f_{n}$ is the frequency of the *n*th harmonic, h is the thickness of the plate, and v is the shear acoustic wave speed, which is related to the material density ρ and the elasticity constant $c_{66}^{D}$. The characteristic resonance frequencies of the TS mode can be used to compute the electromechanical coupling factor $k_{26}$ by:

(5) $$k_{26}^{2}=\frac{\pi }{2}\frac{f_{r}}{f_{a}}\tan\;\left(\frac{\pi }{2}\frac{\Delta f}{f_{a}}\right),$$

where $f_{r}$ and $f_{a}$ are the resonance and antiresonance frequencies, respectively, as annotated on the EMI measurements in Figure 2. Other relevant material constants for the TS mode include the elastic constants $c_{66}^{E}\;$ and $S_{66}^{E}$, the piezoelectric constants $e_{26}$ and $d_{26}$ and the dielectric constant $\epsilon_{22}^{S}$. These parameters are related to the electromechanical coupling factor $k_{26}$, which can be written as:

(6) $$\frac{k_{26}^{2}}{1-k_{26}^{2}}=\frac{e_{26}^{2}}{\epsilon_{22}^{S}c_{66}^{E}}.$$

The elastic constants can be derived using EMI characteristic frequencies, material density and plate thickness, which can be expressed as:

(7) $$c_{66}^{E}=\left(1-k_{26}^{2}\right)\rho f_{p}t^{2},\;c_{66}^{D}=4\rho {\left(tf_{r}\right)}^{2},$$

(8) $$S_{66}^{E}=\frac{1}{4\rho t^{2}f_{a}^{2}\left(1-k_{26}^{2}\right)}.$$

The temperature coefficients of these elastic, piezoelectric and dielectric constants are important for predicting the variation of the frequency response of practical transducers with temperature. The temperature coefficients of the material properties are defined as:

(9) $$T{\left(P\right)}^{\left(n\right)}=\frac{1}{P_{0}n!}{\left(\frac{\delta^{n}P}{\delta \theta^{n}}\right)}_{\begin{array}{c} \theta =T_{0} \end{array}},\;P\left(T\right)=P\left(T_{0}\right)+\sum_{n=1}^{3}T{\left(P\right)}^{\left(n\right)}\cdot {\left(T-T_{0}\right)}^{n},$$

where $T{\left(P\right)}^{\left(n\right)}$ is the *n*th-order temperature coefficient of the material property P, $T_{0}$ is the reference temperature (usually 25 °C), and the property P(T) at temperature T can be calculated using these coefficients.

## 3. Materials and Methods

**Figure 3:**
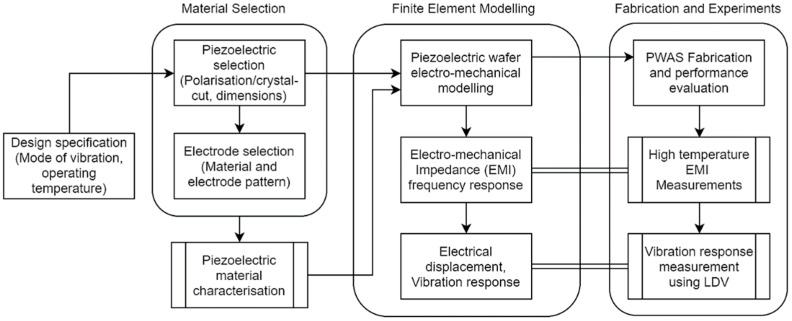
Flow chart showing the methodology followed in this study for transducer design, modelling and validation. Experiments are shown as processes linked to the validation of models.

In this study, a GaPO_4_ plate was examined for the HT guided-wave ultrasonic transducer development. A finite element model was developed to simulate the electromechanical response of the PWAS. The effects of temperature and material degradation on the electromechanical response were evaluated using the model. The modes of vibration within the frequency range of interest for the UGW were also analysed to evaluate suitability and performance as a TS-mode transducer. Both the simulated EMI and the vibration response were validated through experiments. The model development and the experimental setups were described in this section. The methodology followed in this study is highlighted in Figure 3, which shows how the results will be used for a complete transducer design.

### 3.1. GaPO_4_ Piezoelectric Wafer Transducer

**Figure 4:**
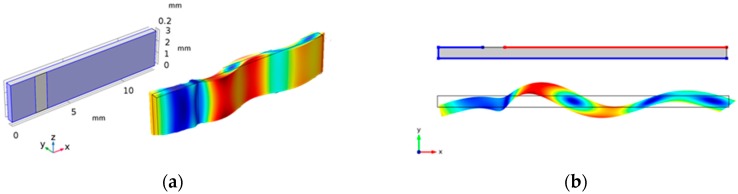
Y-cut GaPO_4_ model geometries and wrap-around electrodes used for 3D (**a**) and 2D (**b**) simulations.

GaPO_4_ resonator plates were obtained by Piezocryst Advanced Sensorics GmBH in a Y-cut (YXl)0° configuration to achieve the desired thickness shear response. The dimensions of the resonator plates were y = 0.5 mm, x = 13 mm and z = 3 mm. These dimensions were chosen to achieve a clean TS mode of vibration for accurate k measurements, as plates with an L/t ratio of <10 lead to a coupling of the TS mode with additional miscellaneous modes [30]. Platinum (Pt) was chosen as the electrode material, as it has excellent conductive properties, resists oxidation and has demonstrated HT performance with no degradation at temperatures up to 650 °C [31,32]. No electrode-matching layer was applied between the two materials, as the CTE of Pt (9 × 10^−6^/°C) matches well with the CTE of GaPO_4_ (12.78 × 10^−6^/°C). Pt electrodes with a thickness of 100 nm were applied using the magnetron sputtering physical vapour deposition (PVD) technique in a wrap-around configuration. The electrode for the ground connection was applied on one of the two large faces, its adjacent end face and part of the second large face, as shown in Figure 4. The electrode for the input voltage was applied on the second large face with a 1 mm gap from the ground electrode, which was achieved by applying a micro-masking tape before sputtering.

### 3.2. Modelling of the GaPO_4_ Transducer

A finite element model of the GaPO_4_ PWAS was developed using the COMSOL Multiphysics (version 5.3a) software package to simulate its piezoelectric behaviour. The piezoelectric device module of COMSOL related the strain and the electric field using Equations (1) and (2). The objectives of this model were to study the impedance resonance spectrum and the modes of vibration within the frequency range of interest for the guided-wave ultrasonic application.

Due to the anisotropic nature of GaPO_4_ (trigonal symmetry), it is necessary to have a full 3D model for accurate estimations, as resonance frequency is highly dependent on crystal orientation (cut angle). However, for a 0°-rotation, Y-cut PWAS, a simpler 2D geometry could be used. Plane strain 2D approximation was used in the model. The results from both the 2D and 3D models were compared, and the TS-mode results were the same. The model geometries and a comparison of the simulated TS-mode shapes are shown in Figure 4. In both cases, the thickness of the PWAS was aligned along the *y*-axis to represent the Y-cut. All the FEA studies in this paper were performed in two dimensions to reduce computation requirements. The dimension, width, was also included as an out-of-plane thickness in the model.

Representative electrical boundary conditions were applied to the PWAS. The 2D geometry in Figure 4 highlights the edges, where the electrical boundary conditions for the ground (blue) and AC terminal (red) voltages were applied. The zero charge condition was applied to all the other edges. Previous studies on the mass loading effect of Pt electrodes have shown that electrodes do not have a significant effect on transducers, where the wavelength is much greater than the electrode thickness [33]. Therefore, in this model, the electrodes were assumed equipotential, i.e., ideal conductor with no atomic mass.

**Figure 5:**
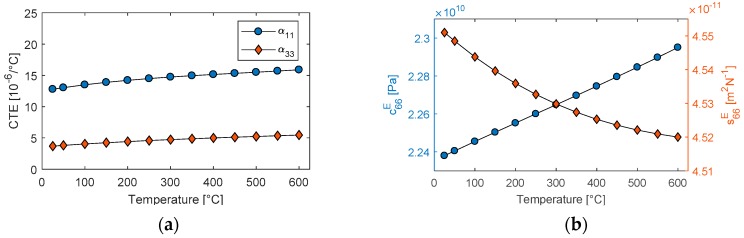
Temperature response of (**a**) coefficients of thermal expansion (CTEs) and (**b**) elastic coefficients, calculated using temperature coefficients from the literature.

The effects of temperature and material degradation were investigated by introducing temperature-dependent material properties of GaPO_4_. The elasticity, piezoelectric and dielectric permittivity matrices and their temperature coefficients were acquired from the material datasheets and the literature [34]. The density at different temperatures was calculated using the following relation:

(10) $$\rho =\frac{m}{lwt}=\frac{m}{l_{0}w_{0}t_{0}\left(1+\frac{\Delta l}{l_{0}}\right)\left(1+\frac{\Delta w}{w_{0}}\right)\left(1+\frac{\Delta t}{t_{0}}\right)}=\;\frac{\rho_{0}}{\left(1+\frac{\Delta l}{l_{0}}\right)\left(1+\frac{\Delta w}{w_{0}}\right)\left(1+\frac{\Delta t}{t_{0}}\right)},$$

where $\rho_{0}$ is the density at room temperature, $\Delta l/l_{0},$ $\Delta w/w_{0}$, and $\Delta t/t_{0}$ were calculated based on the CTEs provided in the datasheet using the following relation. The CTEs $\alpha_{ij}$ of GaPO_4_ is a second-rank tensor with only two independent coefficients $\alpha_{11}$ and $\alpha_{33}$. The temperature coefficients for CTEs and elastic properties were used to compute their respective HT values for the model using Equation (8). The HT material properties are presented in Figure 5.

**Figure 6:**
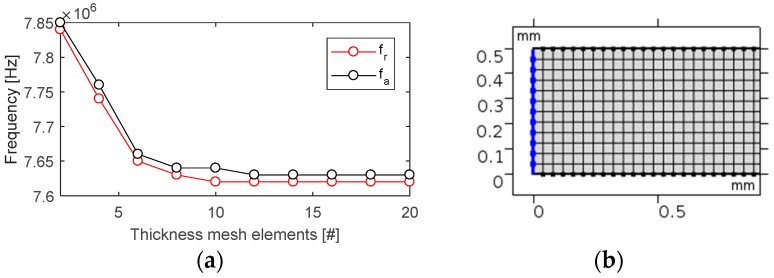
Mesh analysis results showing the change in frequency with an increasing number of mesh elements along the thickness (**a**) and the chosen mesh in the model (**b**).

A fine mapped mesh was utilised in order to achieve a uniform mesh shape throughout the geometry. Previous modelling studies have shown that, for the TS mode, the number of mesh elements along the thickness can have a significant effect on the simulated resonance frequency [35]. A mesh analysis was performed by analysing the characteristic frequencies from the second resonance with an increasing number of mesh elements. Frequencies from the second resonance were chosen, as they are more significantly affected by the thickness compared to the fundamental resonance. From the results in Figure 6, it can be seen that frequency convergence was achieved with 12 mesh elements along the thickness. This related to 14 elements per wavelength at the highest frequency of interest. A mapped mesh with a size of 0.04 mm × 0.04 mm (length × width) was used in all models, which resulted in 3900 mesh elements, covering an area of 6.5 mm^2^.

The model with the optimised mesh was then used to perform two different frequency-domain studies. The first study was conducted within a frequency range of 40 to 12 MHz, to simulate EMI spectra and analyse TS-mode resonance frequencies. The EMI spectrum was extracted using the global evaluation function on the inverse of the admittance parameter (es.Y11) in COMSOL. The second study was performed to evaluate the electrical displacement patterns for the transducer vibrational analysis within a frequency range of 10 to 150 kHz.

### 3.3. Electromechanical Impedance Characterisation

EMI measurements were performed on the GaPO_4_ plates described in Section 3.1. The measurements were performed using a purpose-built test rig [36], which includes a Carbolite LHT6-30 Furnace, and the sample was connected to an Agilent 4294a Impedance/Gain-Phase Analyser through an Agilent 16048A fixture. This test rig was used to evaluate different HT piezoelectric materials, and a comparison of their HT TS-mode properties was reported [37]. The exact dimensions of the samples were measured at an accuracy of 0.01 mm using a digital calliper. Impedance measurements were carried out initially at room temperature and subsequently at temperatures increased with 50 °C intervals up to 600 °C. Before each measurement, the jig was recalibrated by performing a short/open circuit compensation to suppress any parasitic impedance. The impedance spectra were recorded over a frequency range of 40 to 12 MHz with an increment of 15 kHz.

The TS-mode properties were calculated using Equations (3)–(6), where the measured dimensions, the densities of the samples and the frequencies $F_{r}$ and $F_{a}$ were used. For HT values, the properties were calculated using HT dimensions and density. The HT dimensions were calculated by applying Equation (8), and the density at each temperature was calculated using Equations (10). Each of these properties was calculated at several temperatures, and their temperature coefficients were found using curve fitting and the relation defined in Equation (8). The properties and their temperature coefficients were averaged for five samples.

### 3.4. Vibrational Response Measurement

**Figure 7:**
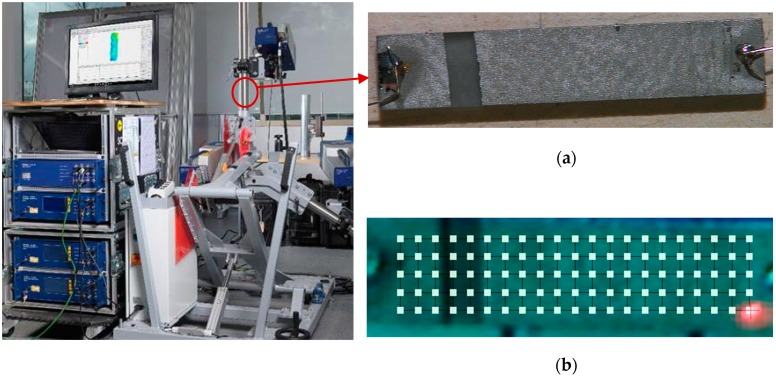
3D laser Doppler vibrometry experimental setup for measuring the vibrational response of the GaPO_4_ piezoelectric wafer active sensor (PWAS) specimen (**a**) showing the scan points used for 3D measurements (**b**).

The ultrasonic vibrational response of the GaPO_4_ PWAS was measured using laser Doppler vibrometry (LDV); the experimental setup is shown in Figure 7. The PWAS were connected to a pulser-receiver unit by soldering thin wires on the Pt-coated surface of the GaPO_4_ plates. A Teletest Focus+ [38] pulser-receiver was used to provide a chirp excitation signal with a frequency range of 10–150 kHz, and a Polytec PSV-400-3D-M scanning laser vibrometer was used to obtain 3D LDV measurements of the GaPO_4_ PWAS surface. The laser beams of the 3D scanner were focused on the surface of the element. 2D and 3D calibration procedures were performed, before the scan points were set to cover the entire surface area of the samples. The collected 3D displacement measurements from all the scan points were averaged and transformed into the frequency domain to evaluate the displacement spectrum.

## 4. Results

### 4.1. Electromechanical Impedance Response

**Figure 8:**
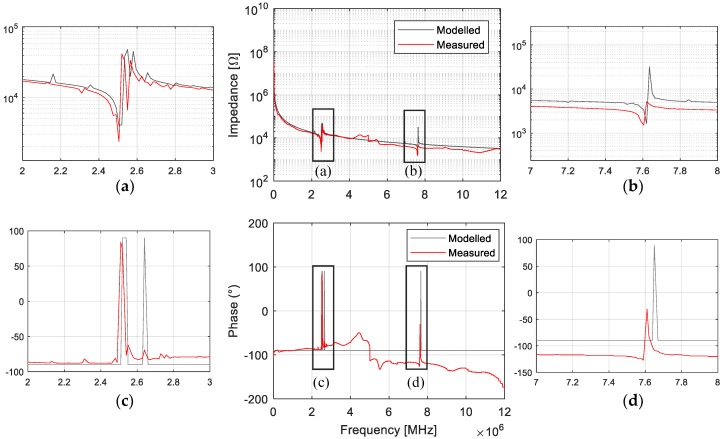
Comparison of the EMI measurements and the modelled responses highlighting impedance amplitude (**a**) and phase (**b**) of the fundamental TS-mode resonance and impedance amplitude (**c**) and phase (**d**) of the overtone.

A comparison of the EMI measurements and the modelled responses of the Y-cut GaPO_4_ plate is shown in Figure 8. Two TS-mode resonances around 2.5 and 7.6 MHz can be observed and were consistent with the frequencies calculated based on Equation (4). A comparison of the calculated, measured and modelled resonances is provided in Table 1. The modelled phase of the impedance was always ±π/2 as in the model, and the resonator was assumed lossless. The coupling to high overtone contours and undesired modes can be seen in the antiresonance frequency of the fundamental resonance, which was also simulated by the model. These unwanted modes usually had lower frequencies, and their influence became smaller for the overtones, as seen in the second overtone.

A slight frequency shift in the measured EMI that affected both resonance and antiresonance frequencies was observed. This was the result of the mass loading effect from the deposited resonator electrode and the connecting wires, which were not included in the model. This is confirmed by the higher disparity in the second resonance, as mass loading is more significant for high overtone resonances, as shown in previous studies [33].

**Figure 9:**
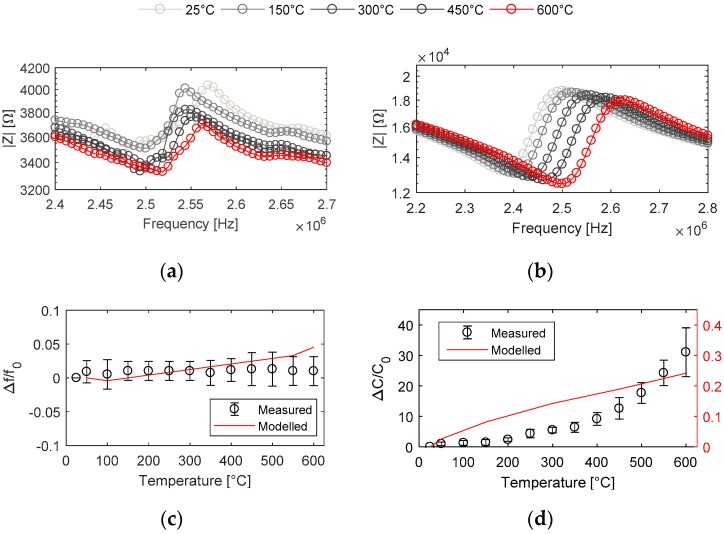
Comparison of EMI response at high temperature: (**a**) measured EMI amplitude; (**b**) modelled EMI amplitude; (**c**) relative change of frequency at temperatures up to 600 °C; and (**d**) relative change in capacitance at temperatures up to 600 °C.

The measured and simulated EMI responses at increasing temperatures, along with its effects on resonance frequency and capacitance, are shown in Figure 9. The measured impedance amplitude at HT was reduced by a factor of 10 compared to that of the response at ambient temperature, and also no mode coupling was observed around the antiresonance frequency. This resulted from the HT conductive adhesive used to attach electrical connection to the PWAS. For the HT models, mechanical damping with an isotropic structural loss factor of 0.01 was added to obtain a comparable frequency bandwidth with the measured response.

With an increase in temperature, a decrease in impedance amplitude and number of antiresonance peaks can be seen. This related to the increase in permittivity and capacitance of the material [39]. Both the measured and simulated EMI responses at HT are in good correlation, which can be seen by a relative shift in resonance and antiresonance frequencies with increasing temperature. The difference in impedance amplitude was associated with the electrical connections within the measurement setup and their parasitic impedance. The temperature coefficient of the resonance frequency predicted by the model was 27.89 × 10^−6^/°C, which matched well with the value of 27.3 × 10^−6^/°C calculated from the measured response with an error of less than 2.2%.

The capacitance data from the model was extracted by computing an integral of the total electric energy on the electrode edge. The increase in capacitance at higher temperatures was observed in both the measured and simulated response. The change in measured capacitance was around 100 times more than that in simulated capacitance. This is due to the combined effect of dielectric losses in GaPO_4_ and parasitic capacitance from the electrical connections and the measurement setup.

### 4.2. TS-Mode Coefficients of GaPO_4_

The TS-mode coefficients were derived from the EMI measurements using Equations (6)–(8). The calculated coefficients were compared with those reported in the literature, and the relative errors are listed in Table 2. The measured elastic coefficients are in good agreement with those reported previously [40]. There was a considerably larger difference in the measured $k_{26}$. This is because of the difference in PWAS geometry, as the previous study used a circular plate with gold electrodes [40]. The deviation was attributed to the electric field concentration at electrode corners in a rectangular design, which was reduced in circular designs. 

The variation between the measured and modelled electromechanical coupling factors $k_{26}$ was less than 3%. This showed that the model can be accurately used to predict the electromechanical resonances and the associated constitutive piezoelectric properties that indicate its performance as an ultrasonic transducer.

**Figure 10:**
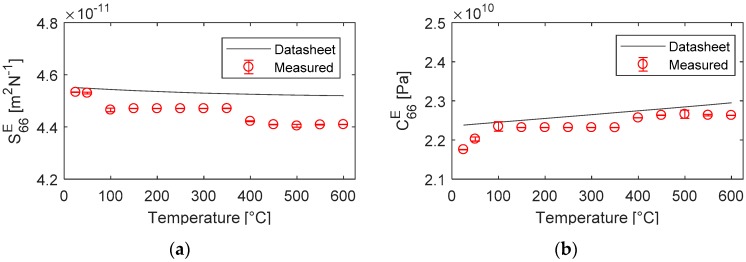
Assessment of the measured elastic constants (**a**) S^E^ and (**b**) C^E^ with those calculated using the temperature coefficients provided in the literature and the datasheets.

The material properties calculated from the HT EMI measurements in Figure 10 were compared with those calculated using the first-, second- and third-order temperature coefficients provided in the datasheet. The temperature dependence of elastic constants exhibited a stable state at temperatures up to 600 °C with a variation of less than 3%. The measured $k_{26}$ was extremely consistent, showing a less than 1% variation at temperatures up to 600 °C. This demonstrated excellent temperature-independent behaviour.

The first-order temperature coefficients were derived from the measured HT properties using Equation (8). The derived coefficients were compared with those available in the literature and are presented in Table 3. A relative error of approximately 3% was observed for the three key FOMs for the TS-mode transducer.

**Figure 11:**
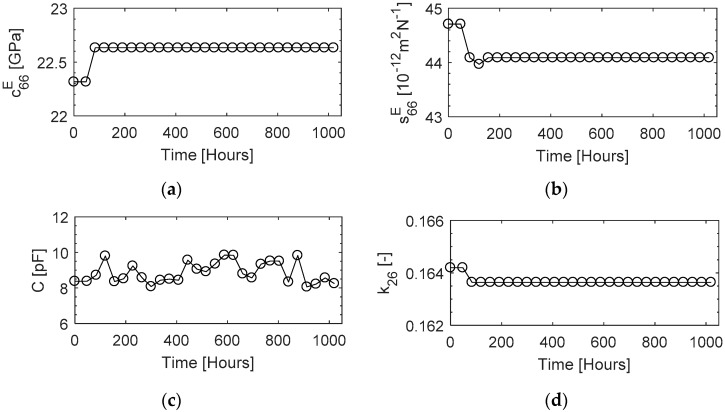
Thermal aging response of the TS-mode elastic coefficients (**a**) C^E^, (**b**) S^E^; (**c**) capacitance; and (**d**) piezoelectric coefficient k_26_ measured at 600 °C for over 1000 h.

The variation in the properties for the estimation of the transducer FOMs was recorded at 600 °C for a period of 1000 hours. The results in Figure 11 show a consistent response from the beginning, with variations of less than 1% in $k_{26}$ and 1.5% in its $s_{66}^{E}$ and $c_{66}^{E}$, which demonstrated an excellent thermal aging behaviour of TS-mode properties, proving its suitability for SHM applications.

### 4.3. Vibration Analysis

**Figure 12:**
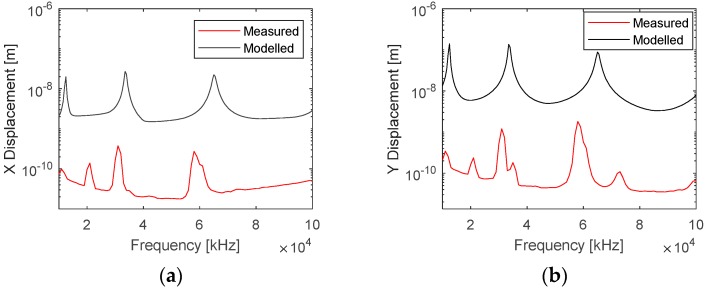
Comparison of the simulated displacement field with LDV measurements in a frequency range of 10–100 kHz showing displacement amplitudes in the (**a**) X and (**b**) Y directions.

A comparison of the vibration response measured using LDV was performed with the simulation of the electric displacement field. The LDV measurements were performed on the length–width plane. The amplitude of the simulated displacement fields in the Y and X directions were extracted from the model using the line average function in COMSOL on the longer edge representing the length–width face of the PWAS. A good correlation between the modelled and measured spectra of the electromechanical displacement field is shown in Figure 12. Three different modes of vibration can be seen with distinct resonance frequencies around 10, 34 and 65 kHz in both the measured and modelled responses. The amplitude of the measured displacement fields was lower than that of the simulated response by a factor of 100. This was associated with friction, dielectric dissipation and losses, which were not considered in the model. A shift in the simulated frequency response was depicted, which increased almost linearly with the increase in frequency. This is possible because of the added mass from electrodes and electrical connections, which were not included in the model. Two other weaker modes were observed at 20 and 70 kHz in the measured response. These were associated to the vibration along the width and were not predicted by the model because of the plain strain 2D approximation.

**Figure 13:**
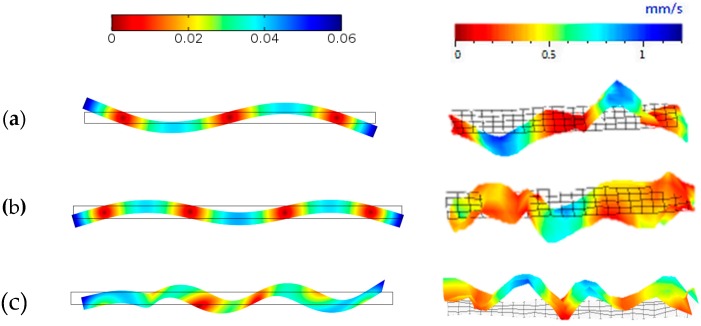
Vibration patterns of the Y-cut GaPO_4_ PWAS simulated (left) and measured (right) for 34 kHz (**a**), 65 kHz (**b**) and 100 kHz (**c**). Colors indicate velocity amplitudes.

Simulated velocity amplitudes from the length–thickness face were derived from the model and compared with LDV measurements. The modelled and measured vibration modes at 34, 65 and 100 kHz were compared and are shown in Figure 13. The modelled and measured results appeared to be in good agreement. The first two vibration modes at 34 and 65 kHz were longitudinal modes. The TS mode can be seen at 100 kHz, as the in-plane displacement was much larger than the out-of-plane displacement. In both the simulated and measured TS modes, a coupling of longitudinal mode can be observed. This is due to the boundary effects of the wrap-around electrode configuration. Acknowledging the knowledge of mode coupling and undesired miscellaneous modes, the model can be used to further study efficient backing materials or alternative electrode configurations to suppress them.

## 5. Discussions

This research focused on the examination of GaPO_4_ for HT applications of UGWs by developing a TS-mode PWAS. An FEA model was developed and used to predict EMI and vibrational response accurately. The thermal response of the simulated EMI was validated with HT experiments and showed good agreement in terms of change in frequency and electromechanical coupling factor, which is a key FOM for ultrasonic applications. The model requires HT coefficients of material properties to simulate piezoelectric losses. If they are not available in the literature, a method for computing these coefficients for the TS mode was presented, which showed a good correlation with properties reported in the literature. To further improve the accuracy of the model, losses such as friction, damping and dielectric dissipation can be extracted from EMI response [42] and added to the model. The 2D model described here can be used to evaluate crystal cuts with no rotation. For single or multiple rotated crystal cuts, either 3D models with rotated coordinate systems or transformation of material properties will be required.

In UGW testing, signal interpretation can be very challenging because of the large number of wave modes that exist in a waveguide. Knowledge of vibration patterns of a transducer and how they are influenced by increasing temperature is crucial for transducer design. The model presented here predicted mode coupling and unwanted contour modes. This demonstrated the suitability of such a technique for optimising transducer design by suppressing unwanted modes.

The model can be further evaluated with time-domain analysis to analyse guided wave excitation on pipelines at HT. This study will require temperature-dependent properties of adhesives used for bonding. This will be useful for analysing excitation of any unwanted mode or transducer failure due to thermal stresses arising in a transducer as a result of differential thermal expansion.

## 6. Conclusions

In this study, GaPO_4_ has been evaluated at temperatures up to 600 °C for UGW applications to superheated steam lines. GaPO_4_ was fabricated into PWAS with dimensions, cut and electrode configuration to obtain the desired TS mode of vibration. The dielectric, elastic and piezoelectric properties corresponding to the TS mode were measured as a function of temperature to derive their temperature coefficients. An FEA model was developed to simulate the vibrational response and the temperature behaviour of the PWAS. These simulation results were validated experimentally with LDV and EMI measurements, respectively, where good agreement with the measured TS-mode shapes and the thermal response was observed. Finally, thermal aging experiments were conducted on the PWAS for over 1000 hours, and a stable TS-mode response was achieved, demonstrating its potential for SHM applications on steam lines. These results are of theoretical importance for the design of HT transducers for UGW applications.

## Figures and Tables

**Table 1:** Comparison of theoretical thickness-shear (TS)-mode frequencies between measurements and simulation.

TS Mode	Frequency (MHz)			Relative Error (%)
	Calculated	Measured	Simulated	
Fundamental (n = 1)	2.53	2.52	2.51	0.4
Overtone (n = 3)	7.59	7.61	7.62	0.13

**Table 2:** GaPO_4_ TS-mode properties at room temperature.

Property	Units	Ref [41]	Measured	Modelled	Relative Error (%)
$C_{66}^{E}$	GPa	22.38	21.76	-	2.85
$C_{66}^{D}$	GPa	23.28	22.36	-	3.95
$S_{66}^{E}$	10^−12^ m^2^N^−1^	45.51	45.33	-	0.4
$k_{26}$	-	0.183–0.192 ^1^	0.164	0.169	7.65

^1^ The reported value is for a different geometry and a different electrode configuration.

**Table 3:** Temperature coefficients of the TS-mode GaPO_4_ elastic and piezoelectric properties.

Property	$Tp_{ij}^{\left(1\right)}\;\left(10^{-6}\;K^{-1}\right)$		Relative Error (%)
	References [40,41]	Measured	
$C_{66}^{E}$	44.9	46.4	3.23
$S_{66}^{E}$	−26.9	−27.8	3.34
$f_{r}$	19.9–35.7 ^1^	27.3	1.8 ^2^
$k_{26}$	452	469	3.62

^1^ Reported values are with and without correction from stray capacitance [41]. ^2^ Error calculated based on the average of the two reported values.
